# Perturbation of Auxin Homeostasis and Signaling by *PINOID* Overexpression Induces Stress Responses in Arabidopsis

**DOI:** 10.3389/fpls.2017.01308

**Published:** 2017-08-02

**Authors:** Kumud Saini, Hamada AbdElgawad, Marios N. Markakis, Sébastjen Schoenaers, Han Asard, Els Prinsen, Gerrit T. S. Beemster, Kris Vissenberg

**Affiliations:** ^1^Integrated Molecular Plant Physiology Research, University of Antwerp Antwerp, Belgium; ^2^Department of Botany, Faculty of Science, Beni-Suef University Beni Suef, Egypt; ^3^Faculty of Health and Medical Sciences, University of Copenhagen Copenhagen, Denmark; ^4^Plant and Biochemistry and Biotechnology Lab, Department of Agriculture, School of Agriculture, Food and Nutrition, Technological Educational Institute of Crete: University of Applied Sciences Heraklion, Greece

**Keywords:** auxin, *PINOID (PID)*, reactive oxygen species (ROS), flavonoids, drought stress, osmotic stress

## Abstract

Under normal and stress conditions plant growth require a complex interplay between phytohormones and reactive oxygen species (ROS). However, details of the nature of this crosstalk remain elusive. Here, we demonstrate that PINOID (PID), a serine threonine kinase of the AGC kinase family, perturbs auxin homeostasis, which in turn modulates rosette growth and induces stress responses in Arabidopsis plants. Arabidopsis mutants and transgenic plants with altered *PID* expression were used to study the effect on auxin levels and stress-related responses. In the leaves of plants with ectopic *PID* expression an accumulation of auxin, oxidative burst and disruption of hormonal balance was apparent. Furthermore, *PID* overexpression led to the accumulation of antioxidant metabolites, while *pid* knockout mutants showed only moderate changes in stress-related metabolites. These physiological changes in the plants overexpressing *PID* modulated their response toward external drought and osmotic stress treatments when compared to the wild type. Based on the morphological, transcriptome, and metabolite results, we propose that perturbations in the auxin hormone levels caused by *PID* overexpression, along with other hormones and ROS downstream, cause antioxidant accumulation and modify growth and stress responses in Arabidopsis. Our data provide further proof for a strong correlation between auxin and stress biology.

## Introduction

The growth regulator auxin plays a role in many aspects of growth and development in plants (Teale et al., 2006; Benjamins and Scheres, 2008; Enders and Strader, 2015). There is increasing evidence for the involvement of auxin metabolism, transport and signaling in stress responses as well (Shibasaki et al., 2009; Shen et al., 2010; Zhang et al., 2012; Kazan, 2013; Remy et al., 2013). Morphogenesis and stress adaptive responses are closely linked to cellular hormonal homeostasis, including auxin. On one hand, mutants overproducing auxin or with altered auxin distribution show developmental defects such as inhibition of shoot growth, cotyledon and leaf epinasty, a longer hypocotyl and excessive lateral root growth (Boerjan et al., 1995; King et al., 1995; Delarue et al., 1998; Zhao et al., 2001). On the other hand, several stress conditions are also known to modify auxin homeostasis and response. Auxin homeostasis could be perturbed by stress-induced changes in the concentration of phenolics compounds such as quercetin and kaempferol (auxin transport inhibitor affecting cycling of auxin efflux carriers), and changes in apoplastic pH affecting auxin uptake and distribution (Potters et al., 2007, 2009). Cadmium treated, phosphorus-, or sulfur-starved plants showed altered auxin metabolism (Potters et al., 2007). Water deficit conditions upregulated Gretchen Hagen 3 (*GH3)* genes, suppressed *YUC* genes and lowered indole-3-acetic acid (IAA) levels in rice (Zhang et al., 2009; Du et al., 2013). Hyper-osmolarity promoted PIN FORMED1 (PIN1) and PIN2 internalization while hypo-osmolarity showed opposite effect in Arabidopsis roots (Zwiewka et al., 2015). Salt stress is also shown to affect free and conjugated IAA levels in the developing xylems in poplar, resulting in negative effects on the expansion of xylem vessels (Junghans et al., 2006). Studies on rice and sorghum suggest that auxin response factors (*ARFs)* and several other early auxin-responsive genes also function in abiotic stress responses (Jain and Khurana, 2009; Wang et al., 2010). Alternatively, stress also induced changes in H_2_O_2_ levels that are co-factors for peroxidases to catalyze the oxidative degradation of IAA (Gazarian et al., 1998; Tognetti et al., 2012). Apoplastic reactive oxygen species (ROS) transiently decreased auxin signaling, modified auxin homeostasis and altered leaf morphology in ozone (O_3_) treated plants (Blomster et al., 2011).

Interestingly, many studies have reported an increase in abiotic stress tolerance in transgenic plants, and in conditions with altered auxin levels (Shi et al., 2014; Naser and Shani, 2016). For instance, elevated auxin levels in transgenic lines overexpressing *YUCCA7* (a gene involved in the auxin biosynthesis pathway) increased resistance to drought stress in Arabidopsis (Lee et al., 2012). Similarly, overexpression of *YUCCA6* led to enhanced resistance to water stress in potato (Kim et al., 2013). Furthermore, ectopic expression of H_2_O_2_-inducible *UGT74E2* led to an increase in free indole-3-butyric acid (IBA, another form of active auxin) and IBA-glucose, and also increased the tolerance to drought stress (Tognetti et al., 2010). Interestingly, Park et al. (2007a), on the other hand, showed that overexpression of the *GH3* gene lowered IAA levels, reduced growth and enhanced the resistance to abiotic stresses. These studies clearly demonstrate a link between auxin physiology and response to environmental stress factors. Clearly, the relation between auxin and stress adaptations is complex. The specific role of auxin in the induction of stress responses also remains elusive.

The kinase PINOID (PID) regulates PIN localizations on the cellular membranes and thus regulates polar auxin transport (PAT; Friml et al., 2004; Kleine-Vehn et al., 2009). *PID* is an early auxin inducible gene and a member of the AGCVIII protein kinase family (Benjamins et al., 2001). AGC kinases are named after three classes of animal proteins involved in a receptor-mediated growth factor signal transduction: protein kinase A (PKA), cyclic GMP-dependent protein kinases (PKG) and protein kinase C (PKC). There are 37 such kinases in Arabidopsis, 23 of which belong to the AGCVIII group (Galván-Ampudia and Offringa, 2007). AGC kinases regulate cell growth, morphogenesis and cell immunity in animals, where mutations and malfunctioning kinases can lead to diseases such as cancer (Pearce et al., 2010). In plants as well, apart from supporting normal development, AGC kinases also participate in stress signaling and in regulation of plant immunity (Devarenne et al., 2006; Hirt et al., 2011; Garcia et al., 2012). For example, the AGC kinase, oxidative signal-inducible 1 (OXI1), is essential for H_2_O_2_ -mediated oxidative stress signaling, defense against the oomycete pathogen *Hyaloperonospora parasitica*, and immunity against *Pseudomonas syringae* (Rentel et al., 2004; Petersen et al., 2009).

We have performed a detailed growth analysis on two *pid* knockouts (*pid-14* and SALK_009478) and two *PID* overexpression lines (*PID^OE^*), 35S::PID10 (addressed here after as P10) and 35S::PID21 (here after P21), and discussed their contrasting effect on auxin homeostasis and rosette phenotypes (Saini et al., 2017). *PID^OE^* lines showed severely reduced rosette growth and high auxin levels in the first pair of leaves. RNA sequencing of the leaves of *PID^OE^* lines pointed toward induction of stress-related responses. Therefore, we here investigate the effect of altered *PID* expression on the perturbation of auxin homeostasis and on the induction of stress responses in whole rosettes in Arabidopsis. We also investigate the effect of such perturbations on the modulation of whole plant responses toward osmotic and drought stress conditions.

## Materials and Methods

### Plant Material and Growth Conditions of Arabidopsis

*Arabidopsis thaliana* Col-0 ecotype seeds were grown in half strength Murashige and Skoog (MS) medium including vitamins (Duchefa, Netherlands), at pH 5.8 containing 1% sugar, 0.5 g/L MES buffer and 0.7% agar. Seeds were sterilized briefly with 70% ethanol followed by 6% bleach for 5 to 10 min and finally rinsed in water. Plants were kept at 21°C in 16/8 h light period under a light intensity of 70–90 μmol m^-2^s^-1^. The knockout T-DNA insertion line SALK_009478 was obtained from NASC. The *pid-14* mutant is SALK_049736 as reported by Haga et al. (2014). The two *PID^OE^* lines, P10 and P21, were developed by Benjamins et al. (2001) by cloning the *PID* cDNA in sense orientation behind the strong Cauliflower Mosaic Virus 35S promoter (35S::PID) and introducing this construct into *Arabidopsis thaliana* ecotype Columbia (Col).

### Drought Stress Application

Seeds (stratified in cold and dark) were sown in separate pots containing an equal amount of soil (Tref substrate). Pots were well watered initially to assist germination. To avoid heterogeneity, trays with pots were rotated 90° on a daily basis. Watering was subsequently withheld 4–5 days after germination. Three conditions were generated, where soil relative water content (RWC) was maintained by weighing pots daily and adjusted to desired values by controlled watering: control pots with 70% RWC, mild stress pots with 45% RWC and severe stress pots at 40% RWC. The experiment continued until 25 days after stratification (DAS). Rosette pictures were taken for measurements with a Canon EOS 40D digital camera equipped with a Canon EF-S 60mm f/2.8 USM macro lens.

For *in vitro* osmotic stress, seeds were sown on half strength MS medium containing different concentrations of mannitol (Duchefa, Netherlands) and Sorbitol (Duchefa, Netherlands) and allowed to grow until 25 DAS. Digital rosette pictures were analyzed using ImageJ^1^.

### Hormone Measurements

To measure concentrations of IAA, abscisic acid (ABA), gibberellins, salicylic acid, cytokinins and jasmonic acid, whole rosettes were harvested at various time points between 7 to 22 DAS. The samples for three replicates were obtained from multiple plates in each experiment. Dissected samples were collected, frozen in liquid nitrogen, and ground using a MagNA Lyser (Roche) with 2 mm glass beads. The detailed hormone extraction procedures and measurements can be found in the Supplementary Material and Methods section.

### qPCR Analysis

Quantitative PCR using SyBr green was performed at 9 and 22 DAS using the first pair of leaves. RNA was isolated using Purelink Plant RNA reagent (Ambion Life Technologies) and quantified with a nanodrop NZ 1000 (Thermo Scientific). An average of 1 μg of RNA was used for first strand cDNA synthesis using RQ1 RNase-Free DNase treatment and the GoScript^TM^ Reverse Transcription System (Promega). Actin 8 primers and gene-specific primers spanning the intron region were used at 55°C as annealing temperature (Supplementary Table 1). A SyBr green assay for qPCR was accomplished according to the developer’s protocol using ROX SYBR Mastermix blue dTTP (Takyon) and a Step one plus Thermocycler (Life technologies). This experiment was done in three independent biological and three technical repetitions. The results were analyzed as ΔΔCT comparison with the StepOnePlus^TM^ Real-Time PCR System (Life Technologies^TM^) software with a confidence level set at 95%.

### RNA Sequencing

18 RNA samples (three genotypes, three replicates, two time points), originating from the first pair of leaves at 9 DAS and 16 DAS from wild type (WT) and both 35S::PID lines were sequenced using an Illumina^TM^ platform. Prior to library preparation the RNA quality and integrity was assessed according to Illumina^TM^ guidelines. Library preparation was done using the TruSeq^®^ Stranded mRNA sample preparation 96-reaction kit (Illumina^TM^) following the low sample protocol according to manufacturer’s recommendations. In brief, approximately 2.5 μg of total RNA was diluted and purified using RNA purification beads targeting the poly-A tail of the mRNA and this was subsequently fragmented by means of the enzymes provided in the kit. After the cDNA synthesis adenylation of 3′ ends and ligation of the adaptors were performed. Adaptors were ligated in 12-plex formations, allowing the pooling of 12 samples. Subsequently, the library was quantified using PicoGreen^®^ dye (Life Technologies^TM^) as described in the manufacturer’s protocol. In order to accurately quantify the concentration in nM of the sample, the Kapa SYBR^®^ FAST universal qPCR kit (Kapa Biosystems^TM^) for Illumina^TM^ sequencing was used to quantify the number of the amplifiable molecules in the sample and the Bioanalyzer^®^ machine (Agilent Technologies^TM^) to determine the average fragment size of the sample. These measurements allowed optimizing the flow cell clustering and proceeding with the Run. The sample was 50 bp pair-end sequenced in one lane of an Illumina^TM^ HiSeq1500 sequencer.

### RNA Sequencing Data Analysis

Resulting sequence data was preliminary analyzed by CLC Genomics Workbench v.6 using the *Arabidopsis thaliana* (Col-0 TAIR10) sequence database^2^ as reference genome. The RNA-Seq analysis was carried out for sequence reads obtained from the three genotypes. Throughout the analysis with CLC default settings were used. Briefly, after the trimming of the sequences they were mapped against the reference genome with the default settings. The expression values were calculated based on “reads per kilo base of exon model per million mapped reads” (RPKM) values (Mortazavi et al., 2008). The RNA-seq data was grouped accordingly and two group comparisons (unpaired) were performed. The expression values were normalized by scaling to the default setting of 10 million reads. Moderated t statistics for pairwise contrasts were calculated using the Baggerly’s test (Baggerly et al., 2003). Genes with no counts in all three replicates for at least one of the genotype/time combinations were discarded as not detectable above the background. Statistical analysis for the effect of genotype and time was conducted by a two-way ANOVA on the MeV software (Multi Experiment Viewer 4.9.0). The Baggerly’s p values were corrected for multiple testing for each contrast separately by means of false discovery rate (FDR; Benjamini and Hochberg, 1995) for significant genes based on ANOVA. FDR corrected *p*-value < 0.05 and log_2_ of fold change > 0.75 was used as a cutoff. All significantly induced or repressed genes with known functions were classified into groups based on gene ontology information obtained from the TAIR Database^3^ using MapMan (Thimm et al., 2004) and overrepresented functions and gene enrichment studies were carried out in Cytoscape using the BiNGO plugin (Maere et al., 2005).

### Metabolite Analyses

Malondialdehyde (MDA) and H_2_O_2_ were determined by the thiobarbituric acid–MDA (TBA–MDA) assay (Hodges et al., 1999) and xylenol orange-based FOX1 method (Jiang et al., 1990), respectively, from a 100 mg frozen sample. Antioxidants were extracted by homogenizing 100 mg sample in 1.4 ml 80% ethanol (v/v). Total antioxidant capacity was measured after mixing the tissue extract with ferric reducing/antioxidant power (FRAP) assay reagent (0.3 M acetate buffer, pH 3.6, 0.01 mM TPTZ in 0.04 mM Hal, 0.02 M FeCl_3_⋅6H_2_O) at 600 nm using a microplate reader (Synergy Mx, Biotek Instruments Inc., Winooski, VT, United States) (Benzie and Strain, 1996). Trolox was used as standard.

### Enzyme Assays

Glycolate oxidase was measured according to Feierabend and Beevers (1972). GO was measured by the formation of a glyoxylate complex with phenylhydrazine (𝜀324 = 17 mM^-1^ cm^-1^). NADPH oxidase (oxidative stress-related enzyme) was assayed according to Sarath et al. (2007) measuring NADPH-dependent superoxide generation as the reduction rate of NBT into monoformazan (𝜀530 = 12.8 mM^-1^ cm^-1^). Enzymes were measured in extracts obtained from 100 mg of frozen tissue, in 1 mL of extraction buffer: 50 mM MES/KOH (pH 6.0) containing 0.04 M KCl, 2 mM CaCl_2_ and homogenized with a MagNA Lyser (Roche, Vilvoorde, Belgium). Catalase activity was assayed by observing the H_2_O_2_ decomposition rate (𝜀240 = 39.4 M^-1^cm^-1^) (Aebi, 1984). SOD activity was determined according to Dhindsa et al. (1982) by measuring the inhibition of NBT reduction at 560 nm. SOD and CAT measurements were scaled down for semi-high throughput measurement using a micro-plate reader (Synergy Mx, Biotek Instruments Inc., Winooski, VT, United States). Glutathione *S*-transferase (GST) activity was determined according to Habig et al. (1974). Enzyme activity was calculated by measuring the conjugation of GSH with excess 1-chloro-2,4-dinitrobenzene (CDNB) in 50 mM potassium phosphate buffer at 340 nm. The activity of glutaredoxin enzymes was measured according to Holmgren and Aslund (1995). The assay contains tris-HCl buffer (100 mM, pH 8.0), 100 μg/mL bovine serum albumin, 1 mM GSH, 6 μg/mL yeast glutathione reductase, 0.1 M Tris-Hcl, 2 mM EDTA, 0.4 mM NADPH and 0.7 mM of 2-hydroxy-ethyl-disulfide (HED). The peroxiredoxin activity was performed by measuring the decrease in H_2_O_2_ concentration in the reaction mixture contained 100 mM K_2_HPO_4_ buffer (pH 7.0), 0.3 to 3 mM Prx, 10 mM DTT, and 100 uM H_2_O_2_ (Horling et al., 2003). The reaction was stopped with 12,5% trichloroacetic acid (TCA). To quantify the reduction in H_2_O_2_ content, 10 mM Fe(NH4)_2_(SO_4_)_2_ and 2.5 mM KSCN were added and the absorbance was measured at 480 nm. Ferredoxin enzyme activity measured in a 50 mM HEPES-KOH (pH 8.0) contains 5 mM MgCl_2_ 0.3 mM NADP^+^, 3 mM glucose 6-phosphate, 1 unit/mL glucose 6-phosphate dehydrogenase and 1 mM potassium ferricyanide. The decrease in NADP^+^ was measured at 420 nm.

### Superoxide Detection in Leaves

Superoxide radical detection was performed using the nitroblue tetrazolium (NBT) staining method (Ramel et al., 2009). NBT reacts with O^2-^ to form a dark blue insoluble formazan compound. Arabidopsis plants were infiltrated in 0.1% (w/v) NBT solution in PBS buffer (0.2 g KCl + 0.2 g KH_2_PO_4_ + 1.15 g NaH_2_PO_4_ + 8 g NaCl in 1000 ml of H_2_O; pH 7.1) containing 10 mM of sodium azide (NaN_3_), for 30 min under vacuum. After 4 h of incubation they were cleared and pictured under a Nikon AZ-100 macroscope equipped with a Nikon DS-Ri1 digital camera.

### Statistical Analysis

Rosette and auxin measurements were analyzed by *t*-test using the R statistical package^4^. Conditions of normality of distribution and homogeneity of variance were checked and met. Metabolite and enzyme data were analyzed using SPSS (SPSS Inc, Chicago, IL, United States). Conditions of normality of distribution and homogeneity of variance were not examined due to the small sample size. One-way analyses of variance (ANOVA) were performed on the original data to evaluate the differences between genotypes and time (days). Significant differences between means were determined with the Duncan test (*P* < 0.05).

### Accession Number

The Arabidopsis Genome Initiative locus identifier for the *PID* gene is AT2G34650. Raw RNA sequencing data were deposited in NCBI’s Gene Expression Omnibus as GEO accession no. GSE82086.

## Results

### Overexpression of *PID* Leads to Smaller Rosettes and Elevated Auxin Pools

**FIGURE 1:**
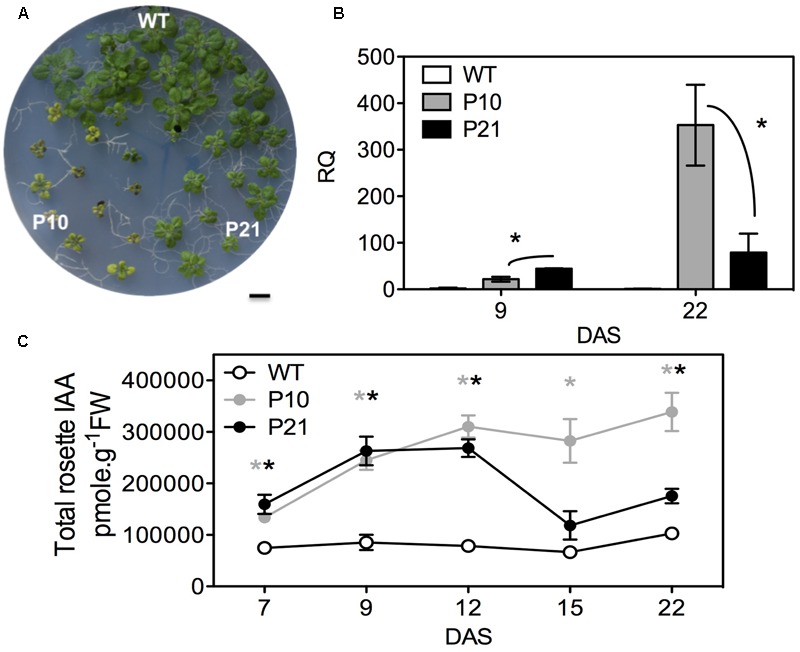
Effect of *PID* overexpression on rosette phenotype and auxin pool in the whole rosettes. Rosette phenotypes of WT and two PID*^OE^* lines, P10 and P21, at 22 DAS **(A)**. Relative quantification (RQ) of *PID* transcript in the leaves of WT, P10, and P21 at two different time points, *n* = 3; Asterisks mark the significant differences between the *PID* overexpression (*PID^OE^*) lines **(B)**. Total auxin (indole-3-acetic acid; IAA) measurements in the rosettes **(C)**. The averages and standard error of relative expression are based on three biological replicates. Gray and black asterisks represent significant differences toward WT, for P10 and P21, respectively. DAS, days after stratification. Error bars: ±SE (*t*-test *P* < 0.05). Scale bar = 10 mm.

In an attempt to deepen our knowledge on auxin-regulated leaf growth and development, we chose to analyze two *PID^OE^* lines, P10 and P21, which clearly had smaller rosettes compared to WT at 22 DAS (days after stratification; **Figure 1A** and Saini et al., 2017). To quantify the *PID* transcript levels between WT and *PID^OE^* lines and to understand the differences in the phenotype between P10 and P21, we performed qPCR. Relative quantification of *PID* transcript levels revealed that P21 had a two-fold higher *PID* overexpression level than P10 at 9 DAS, whereas at 22 DAS this was inversed and *PID* overexpression in P21 was four-fold lower compared to P10 (**Figure 1B**). The severity of the phenotype and *PID* transcript abundance differences between P10 and P21 suggests that PID affects rosette growth in a dose-dependent manner (**Figures 1A,B**). Since PID affects auxin transport and thus its distribution (Christensen et al., 2000; Raftopoulou, 2004; Kleine-Vehn et al., 2009), we measured the total pool of IAA (free IAA and IAA-conjugates). IAA levels were strongly elevated in the rosettes of both *PID^OE^* lines compared to the WT from 7 to 22 DAS (**Figure 1C**). Significantly higher IAA levels in P10 and P21 from 12 DAS onward reflect the difference in expression levels and that a certain threshold of *PID* abundance is required to sustain these high IAA levels (**Figures 1B,C**).

### Transcriptome Data Point to the Induction of Stress in *PID* Overexpression Lines

**FIGURE 2:**
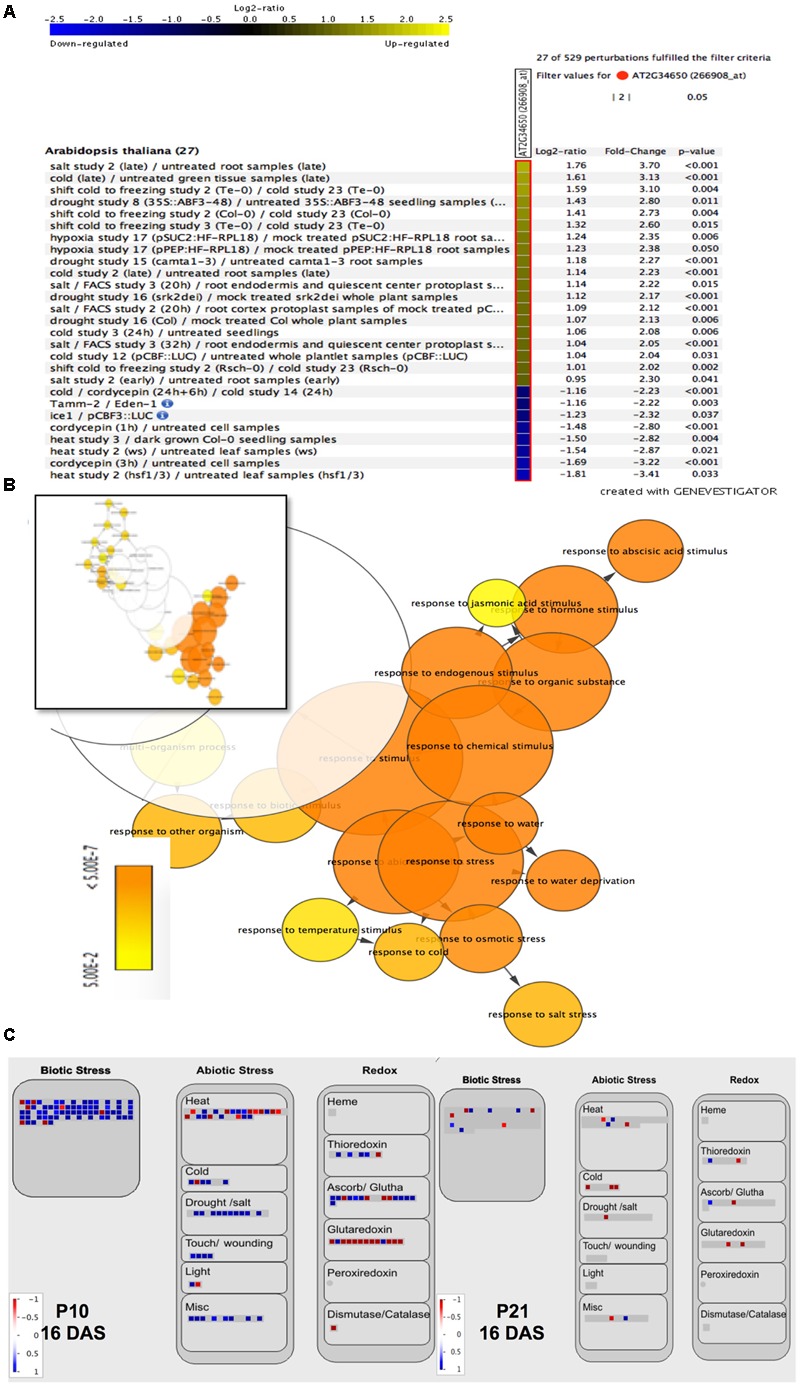
Effect of abiotic stress on *PID* expression levels and of *PID* overexpression on stress-related genes. Genevestigator analysis shows up- (yellow) and downregulation (blue) of *PID* under various abiotic stress conditions **(A)**. Gene enrichment analysis of differentially expressed genes in *PID* overexpression lines vs. WT, visualized via the Cytoscape plugin BiNGO. The inset shows a representation of all 3805 genes, while the major overrepresented genes (orange part of the inset) are magnified **(B)**. MapMan analysis of up- (blue squares) and downregulated (red squares) genes in P10 and P21 at 16 DAS compared to WT, shows the upregulation of biotic and abiotic stress-related genes and changes in redox status **(C)**.

To gain further insight into PID function, we checked the expression levels of the AT2G34650 gene (*PID*) under various conditions in Arabidopsis using perturbation as condition search tool in Genevestigator. Publicly available microarray data revealed that *PID* is differentially expressed in response to several abiotic stress conditions (**Figure 2A**). We performed RNA sequencing on the first pair of leaves of *PID^OE^* lines and WT at 9 and 16 DAS to understand the small-rosette phenotype (Supplementary Data 1). In total there were 3805 genes that expressed differentially in at least one genotype and one time point (FDR < 0.05 and log_2_ fold change > 0.75; Supplementary Data 2). Consistent with the link to abiotic stress suggested by Genevestigator, gene enrichment analysis of the 3805 differentially expressed genes (DEGs), using BiNGO (Maere et al., 2005), showed a significant overrepresentation of ABA signaling, abiotic and biotic stress-related genes, stimulus to chemical and stress, including response to water, water deprivation and osmotic stress (**Figure 2B** and Supplementary Data 3). Moreover, a MapMan (Thimm et al., 2004) plot of the DEGs also showed that many stress and redox-related genes were among induced or repressed genes in P10 and P21 (**Figure 2C**). The MapMan analysis also revealed that similar categories were changed in P10 and P21, but at a more pronounced level (more genes and higher fold changes) in P10. This is consistent with the *PID* expression level of P10 being higher than in P21 at 16 DAS when the transcriptome analysis was performed (**Figure 1B**).

### Multiple Hormonal Responses Are Modulated in *PID* Overexpression Plants

Physiological and molecular mechanisms underlying plant growth and stress responses are complex and likely involve feedback mechanism and crosstalk with other hormones. Consistently, several auxin metabolism, transport and signaling-related genes were affected by *PID* overexpression that point at a perturbed auxin homeostasis and signaling. These include *GH3*, IAA-leucine conjugate hydrolase (*ILR1*), indole-3-butyric acid response (*IBR 1* and *2)*, PINs, auxin/indole-3-acetic acid (Aux/IAAs), small auxin up RNAs (SAURs), Lateral Organ Boundaries-Domain (LBDs), and auxin responsive factors (ARFs) (Supplementary Data 2). Auxin is known to modulate the metabolism of other hormones (Nemhauser et al., 2006). In our data, besides auxin, other hormone signaling components were also modulated in the overexpression lines (Supplementary Figure 1). The expression level of signature genes of several hormones (Nemhauser et al., 2006) were significantly affected in at least one of the *PID^OE^* lines on one time point: ABA-induced carotenoid dioxygenase (*NCED3*) and abscisic acid responsive elements-binding factor (*AREB1/ABF2, AREB2/ABF4*, and *ABF3*), gibberellic acid (GA) inducible, *RGL1*, and jasmonic acid induced, lipoxygenases *LOX1* and *LOX2* were all upregulated, whereas cytokinin-induced response regulators, *ARR4* and *UGT76C2*, were downregulated (see Supplementary Table 2 for full list). Thus, these data suggest that the accumulation of IAA in the *PID^OE^* lines leads to changes in signaling components of other hormones as well. Consistently, there was an overrepresentation of stress-related genes that are reported to function in ABA-dependent stress responses. This includes ABA insensitive (*ABI1* and *ABI2*), *AREB1/ABF2* and *AREB2/ABF4* and *ABF3* (Murata et al., 2001; Abe et al., 2003; Fujita et al., 2005; Yoshida et al., 2010). Multiple genes involved in stress regulation that are auxin inducible, such as *RAB18, RD22, RD29A, RD29B* (Shi et al., 2014; Supplementary Table 2), were also differentially expressed in our data, suggesting upregulation of hormone-related stress responses in *PID^OE^* lines.

**FIGURE 3:**
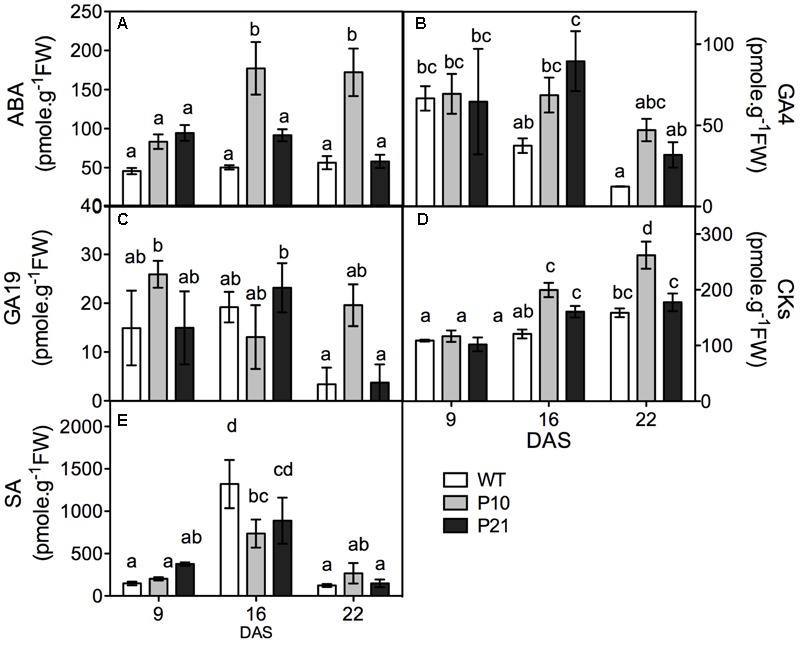
Hormone quantification in the rosettes of WT and *PID* overexpression lines at different time points. Abscisic acid (ABA; **A**). Gibberellic acid (GA4 and GA19; **B,C**). Cytokinins (CKs; **D**) and salicylic acid (SA; **E**). Different letters in the graph represent significant differences between genotypes and time. (Duncan test; *P* < 0.05; *n* = 3; Error bars: ±SE).

To study whether indeed the levels of other hormones were affected in the *PID^OE^* lines, we quantified ABA, GA, cytokinins, salicylic acid, and jasmonic acid in the rosettes (**Figure 3**). Both *PID^OE^* lines showed elevated ABA levels at 9 and 16 DAS compared to the WT, while P10 also maintained significantly high ABA levels at 22 DAS (**Figure 3A**). Thus ABA concentration varied in parallel with *PID* expression levels and the auxin accumulation. *PID^OE^* lines had slightly increased GA4 levels at 16 DAS, while there was a clear increase in cytokinins levels at 16 and 22 DAS. Salicylic acid and GA19 levels were not significantly changed between the genotypes (**Figures 3B–E**), whereas other GAs were either absent or below the detection limit. Also jasmonic acid concentrations were below the detection limit (1.41 pmol/g).

### *PID* Overexpression Lines Show Changes in the Activity of Stress Markers

**FIGURE 4:**
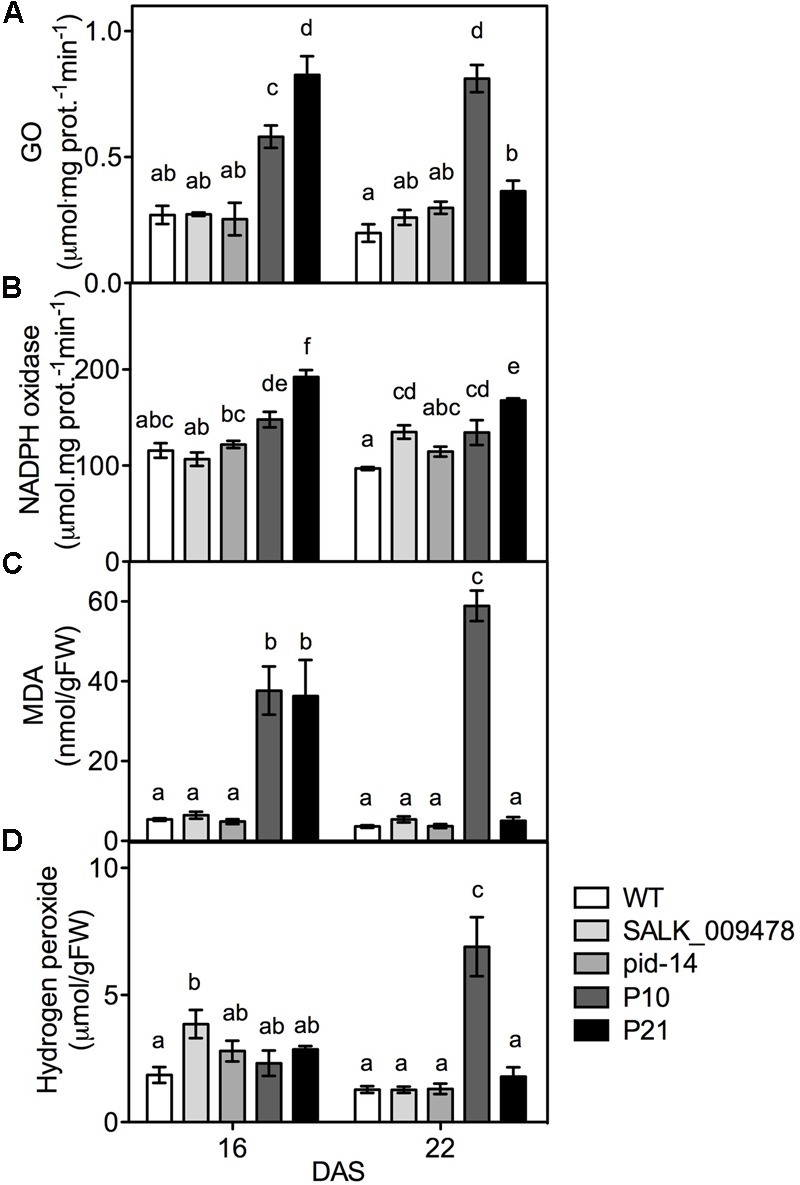
Quantification of oxidative stress indicators in rosettes of WT and lines with altered *PID* expression. Glycolate oxidase; GO activity **(A)**, NADPH oxidase activity **(B)**, malondialdehyde; MDA **(C)**, and hydrogen peroxide abundance **(D)** in the rosettes of WT, *pid* knockouts and *PID^OE^* lines at 16 and 22 DAS. Different letters in the graphs (a–f) represent significant differences between genotypes and time (Duncan test; *P* < 0.05; *n* = 3; Error bars: ±SE).

To verify the transcriptome data and study the relation between stress and *PID*, we tested the effect of altered *PID* expression on cellular stress markers using both *pid* knockouts and overexpression mutants. Being a common response to most adverse abiotic conditions, we looked at the parameters related to oxidative stress (Polle and Rennenberg, 1993). Photorespiration, NADPH oxidase, apoplastic peroxidases, acyl-CoA oxidase, and mitochondrial electron transport are among important ROS sources and some have also been involved in responses related to IAA (Elstner, 1982; Wingler et al., 2000; Gill and Tuteja, 2010; Sandalio et al., 2016). We measured the parameters that were also differentially expressed in our transcriptome data (Supplementary Table 3). Our global transcript data suggest an upregulation of photorespiration (Supplementary Table 3), and both *PID^OE^* lines showed elevated GO activities at 16 DAS and P10 at 22 DAS (**Figure 4A**). The elevation in GO activity was closely linked to *PID* expression levels (**Figure 1B**). Compared to the WT, NADPH oxidase activity was increased in both the *PID^OE^* lines at 16 and 22 DAS (**Figure 4B**). Unlike in the *PID^OE^* lines, the activities of both enzymes were largely unchanged in *pid* mutants relative to the WT. Oxidative damage to the cell membranes can be measured in terms of MDA levels. Our results show increased MDA levels in the overexpression lines, but these were unchanged in *pid* mutants (**Figure 4C**), clearly suggesting lipid peroxidation in the membranes of *PID^OE^* lines, unlike in the membranes of the WT and *pid* knockouts. H_2_O_2,_ quantified using the xylenol orange-based FOX1 method (Jiang et al., 1990), showed a gradual increase from 16 to 22 DAS to six-fold higher levels in P10 at 22 DAS, while the mutants and P21 showed no differences compared to the WT (**Figure 4D**). Rosettes and leaves from all the three genotypes were stained for superoxides and both P10 and P21 showed high staining based on the reaction between NBT and O^2-^, leading to the formation of the blue colored formazan compound. The staining was most prominent at 9 DAS and particularly enriched in the top-half of the leaves of *PID^OE^* lines, while it was spread across the leaf blade in the WT suggesting a relation between auxin accumulation (i.e., top leaf blade in *PID^OE^* lines) and superoxide radicals (Saini et al., 2017; Supplementary Figure 2).

### *PID* Overexpression Lines Have High Cellular Antioxidant Levels

**FIGURE 5:**
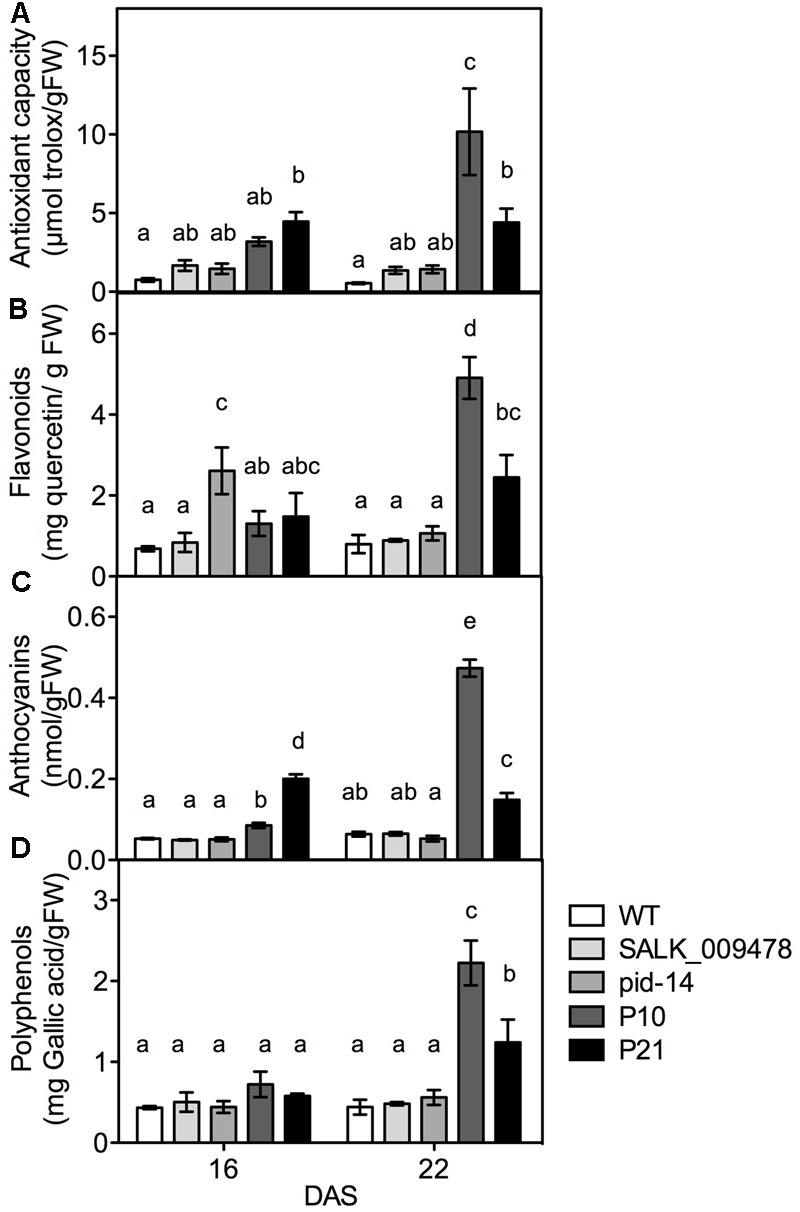
Quantification of molecular antioxidants and related enzymes in rosettes of WT and PID overexpression lines. Total antioxidant capacity; TAC **(A)**, flavonoids **(B)**, anthocyanins **(C)**, and polyphenols **(D)** in WT and *PID^OE^* lines at 16 and 22 DAS. Different letters in the graph represent significant differences between the genotypes and time (Duncan test; *P* < 0.05; *n* = 3; Error bars: ±SE).

Given the dual role of ROS as signaling molecules and molecules causing cellular damage, plants strictly regulate ROS levels in various cellular compartments by means of various antioxidant systems (Noctor and Foyer, 1998; Arora et al., 2002; Neill et al., 2002; Foreman et al., 2003; Foyer and Noctor, 2005). Therefore, we determined the concentrations of some chemical antioxidants and enzymatic antioxidants in *pid* mutants, *PID^OE^* lines and the WT. The transcripts related to phenylpropanoid and flavonoid biosynthesis pathways showed higher abundance in *PID^OE^* lines compared to the WT in the transcriptome data (Supplementary Figure 3 and Data 2). Since the results could indicate higher concentrations of the end products, we measured the concentrations of flavonoids and anthocyanins along with the total antioxidant capacity in all the genotypes. Indeed, the total antioxidant capacity, reflecting overall changes in the radical scavenging capacity, was higher in P21 lines at 16 DAS and in both lines at 22 DAS compared to the WT (**Figure 5A**). In addition to the enhanced total antioxidant capacity, also the levels of one or several groups of antioxidant molecules increased gradually (over time, from 16 to 22 DAS) and considerably in at least one genotype and time point in *PID^OE^* lines. This is particularly clear for flavonoids, anthocyanins, and polyphenols, more so in P10 than P21 (**Figures 5B–D**), which is again in congruence with comparatively higher auxin levels, *PID* transcript and ROS accumulation in P10 (**Figures 1, 4D**). Contrary to *PID^OE^* lines, antioxidants levels were unchanged in *pid* knockout mutants compared to the WT, with exception of increased flavonoids in *pid-14* at 16 DAS. At the level of enzymatic antioxidants, with the exception of catalase (CAT), a large number of enzymes showed moderate changes in *pid* knockouts and overexpression lines [superoxide dismutase (SOD), peroxidases (POX), glutaredoxins, ferredoxins, and peroxiredoxins), while levels of GSTs, enzymes involved in detoxification of xenobiotic compounds (Marrs, 1996), were unchanged in all the genotypes (Supplementary Figure 4). These results demonstrate an altered antioxidant status as a consequence of altered *PID* expression.

### *PID* Overexpression Lines Show Modulated Response to External Stresses

**FIGURE 6:**
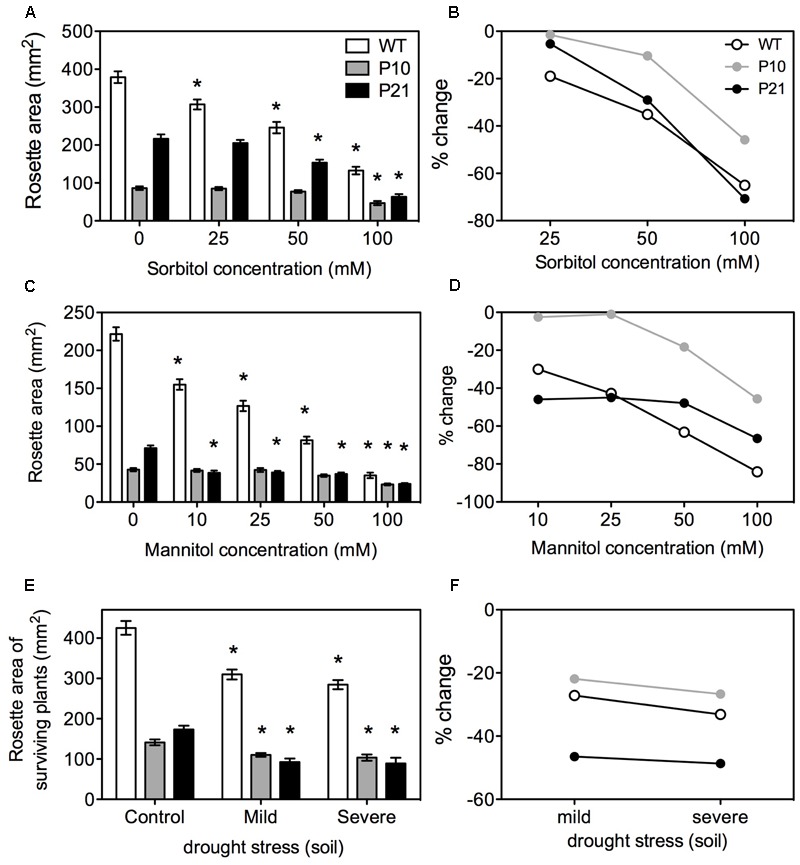
Effect of osmotic and drought stress on rosettes of WT and *PID* overexpression lines. Rosette area under increasing concentrations of sorbitol at 25 DAS; Error bars: ±SE **(A)** and corresponding percent changes; Error bars represent error of propagation **(B)**. Rosette area under increasing concentrations of mannitol at 25 DAS; Error bars: ±SE **(C)** and corresponding percent changes; Error bars represent error of propagation **(D)**. Rosette area measurements under soil drought stress at 25 DAS (Error bars: ±SE) with percent changes **(E,F)**. Asterisks represent statistical significant differences with the respective controls (*n* = 40–50; *P* < 0.05).

Many reports showed that modulation of auxin and antioxidant levels confer resistance or tolerance to drought or osmotic stress (Tognetti et al., 2010; Lee et al., 2012; Espinoza et al., 2013; Kim et al., 2013; Cha et al., 2014; Nakabayashi et al., 2014; Shi et al., 2014; Avramova et al., 2015, 2017; Islam et al., 2015). To investigate if the PID-mediated auxin responses in stress regulation affected whole plant responses to abiotic stresses, we subjected *PID^OE^* lines to osmotic and drought stress. Mannitol and sorbitol were used to lower the water potential in the media (Verslues et al., 2006; Claeys et al., 2014). Rosette area measurements were made at 25 DAS when the first pair of leaves have reached maturity (Beemster et al., 2005). Increasing concentrations of sorbitol or mannitol reduced rosette growth in WT (**Figures 6A–D**). All the lines showed growth reduction although both *PID^OE^* lines, especially P10, showed less impact of the water deficit.

To further confirm the *in vitro* responses to low water potentials, plants were subjected to different water availabilities in soil by altered watering regimes. WT, P10, and P21 showed a growth reduction under mild (45% RWC) and severe (40% RWC) drought stress. Growth reductions in P10 were similar to WT and stronger in P21 (**Figures 6E,F**). After 4 weeks, WT plants had a 100% survival rate (i.e., number of plants surviving at 25 DAS), while the survival in P10 and P21 was reduced (90 and 80% in mild stress; 55 and 35% in severe stress, respectively).

## Discussion

### *PID* Overexpression Perturbs the Homeostasis of Auxin and Other Hormones, Inducing Stress Responses in Arabidopsis Rosettes

Previously, we elaborated on the fact that *PID* overexpression causes reduced growth in Arabidopsis leaves due to elevated auxin levels. On the other hand, *pid* knockouts displayed similar rosette growth and total auxin levels as the WT (Bennett et al., 1995; Christensen et al., 2000; Saini et al., 2017). In order to get a better understanding of the molecular changes induced by the altered *PID* expression levels we performed RNA sequencing on WT and *PID^OE^* lines. Our transcriptome data suggested differential expression of genes related to auxin metabolism and signaling in *PID^OE^* lines (Supplementary Figure 1 and Data 2). Since PID is a regulator of PAT (Benjamins et al., 2001) and controls the subcellular localization of PIN proteins (Friml et al., 2004), the most likely explanation for the increased auxin levels is due to defective transport from the young leaves, which are generally considered as a site of auxin production (Ljung et al., 2001; Saini et al., 2017).

Several studies using genetic mutants with modified auxin metabolism or signaling have demonstrated a role for auxin in abiotic stress. Such studies have, so far, mostly assessed the plant’s tolerance toward abiotic stresses. Here, we uniquely show that PID, a regulator of auxin transport, when overexpressed in Arabidopsis perturbs auxin homeostasis, which consequently leads to induction of stress responses in the rosettes (measured in terms of severity of phenotype, ROS, antioxidants accumulation and upregulation of genes known to be involved in stress and redox signaling in plants; Supplementary Data 2, 3). It is evident from public transcriptome data that *PID* is differentially expressed in stress conditions, although up to four-fold only (**Figure 2**), suggesting that PID might play a role in stress responses. However, a role for PID as a regulator of PIN polarity and thus, auxin transport, is well established and we believe that it is the auxin accumulation in the leaves that causes the reduction in rosette growth, induction of stress responses and modification of plant response toward drought treatment. Our study does strongly point to a relationship between PID-modulated auxin homeostasis and signaling, and the observed stress responses.

*PID^OE^* plants also showed changes in levels of other hormones and their responsive genes, as shown by PageMan, hormone quantification and expression changes in our transcriptome data (**Figure 3**, Supplementary Figure 1, and Data 2). The involvement of multiple hormones suggests involvement of complex and interlinked hormonal regulatory pathways in growth and stress responses in Arabidopsis leaves, that is also frequently evidenced across literature (Mouchel et al., 2006; Nemhauser et al., 2006; Chapman and Estelle, 2009; Harrison, 2012; Lanza et al., 2012; Šimášková et al., 2015). Several reports indeed suggest that auxin conjugates could be involved in stress responses (Tognetti et al., 2010; Ludwig-Müller, 2011; Kinoshita et al., 2012). In fact a stress responsive *GH3* gene, *WES1*, is known to modulate the cross talk between auxin-SA and auxin-ABA (Park et al., 2007a,b; Zhang et al., 2007). Upregulation of *MYB96*, a molecular link in the ABA-auxin crosstalk in stress conditions, along with other genes involved in the *MYB96* regulated pathways such as *RD-22* (drought stress inducing gene) and *GH3* suggests the involvement of auxin-ABA interactive responses in the *PID^OE^* lines (Seo et al., 2009). However, since PID is only known to directly affect auxin, we primarily focus on auxin and speculate that other hormones act downstream of auxin or in concert with auxin to induce stress responses.

**FIGURE 7:**
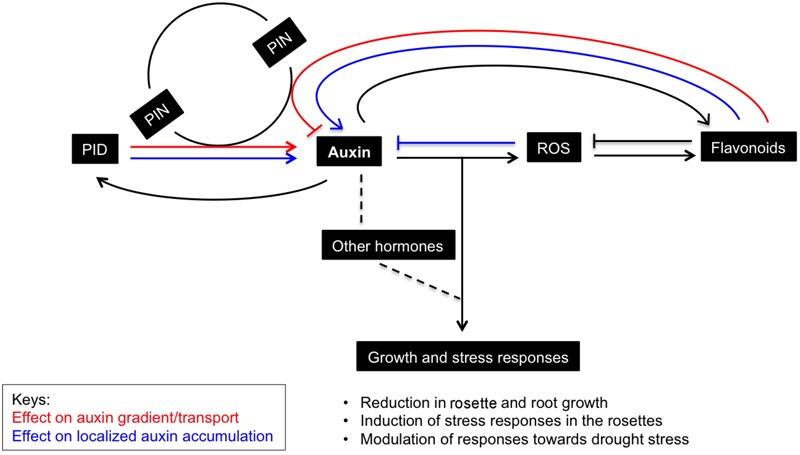
Schematic representation of the effects of PID-modulated auxin homeostasis and signaling on rosette growth and stress responses. *PID*, an early auxin responsive gene and a regular of PIN polarity and auxin transport, causes accumulation of auxin in the rosettes when it is ectopically expressed. The perturbation in auxin homeostasis and signaling affects levels of other interacting hormones and leads to ROS generation, which is known to promote oxidative degradation of auxins. Consequently, flavonoids accumulate to curb ROS and inhibit auxin transport, and supposedly further promote auxin retention in the leaves. This results in growth retardation and increased sensitivity toward drought stress in Arabidopsis plants. Factors affecting the auxin gradient and localized auxin accumulation (here in leaves) are marked in red and blue, respectively.

Like hormones, ROS are signaling molecules that co-regulate growth and development in plants and coordinate responses to environment cues (Neill et al., 2002; Mittler et al., 2004; Jaspers and Kangasjärvi, 2010). Plants respond to environmental stresses by adopting various developmental modulations that include altered growth and development (collectively known as stress induced morphogenic responses; SIMR), reduced metabolism and increased antioxidant accumulation. In fact, plant growth is influenced by a controlled balance between ROS and hormones where auxin and ROS are seen as the key players in stress adaptive responses (Potters et al., 2007, 2009; Tognetti et al., 2012). Evidences showing frequent crosstalk between ROS signaling and hormonal networks are not uncommon. Interplay between ROS and phytohormones is evidenced in abiotic stress adaptation (Tognetti et al., 2010), induction of plant defense responses (Mühlenbock et al., 2008), programmed cell death (Kuriyama and Fukuda, 2002; Gechev et al., 2004), growth and developmental aspects such as cell cycle and cell elongation (Hirt, 2000; D’Haeze et al., 2003; Teale et al., 2006) and regulation of stomatal aperture (Neill et al., 2002). Auxin induced changes in ROS levels are shown to facilitate root gravitropism (Joo et al., 2001), stomatal opening (Song et al., 2006) and cell elongation (Schopfer, 2001). Several sources of ROS, i.e., NADPH oxidases, photo respiratory enzymes, apoplastic peroxidases, acyl-CoA oxidase, and mitochondrial electron transport, are also involved in IAA regulatory networks (Sandalio et al., 2016). *PID^OE^* lines had elevated ROS levels that can possibly be explained by increased photorespiration, but also by increased NADPH oxidase activity (Peer et al., 2013). Auxin overproducing/accumulating mutants are known to have reduced rosette growth and epinastic leaves (Boerjan et al., 1995; Delarue et al., 1998; Zhao et al., 2001). Leaf epinasty is primarily controlled by auxin and involves ROS-auxin interplay as well (Sandalio et al., 2016). *PID^OE^* lines also show enhanced auxin and ROS and downward leaf curling (Christensen et al., 2000; this study). We believe that the high auxin levels in *PID^OE^* lines could be causing high ROS production, resulting in growth retardation and distinct morphological phenotypes in the leaves (**Figure 7**).

Apart from growth reduction, plants adapt to abiotic stress by mechanisms such as stomatal closure, accumulation of osmolytes, antioxidants, and the induction of LEA proteins (Verslues et al., 2006). Similarly, in response to induced stress conditions due to *PID^OE^*, various antioxidants appeared to buffer ROS accumulation in plants. The increase in overall antioxidant capacity is probably caused by increase in flavonoids, anthocyanins, and other polyphenols. Consistently, most of the genes related to flavonoids/anthocyanin biosynthesis were also upregulated in the *PID^OE^* lines (Supplementary Figure 3 and Data 2). Flavonoids and anthocyanins are plant secondary metabolites produced via the phenylpropanoid pathway that affect several developmental processes including protection against UV, ROS, etc. (Taylor and Grotewold, 2005; Grotewold, 2006). Flavonoids are known to alter auxin transport and promote localized auxin accumulation in a tissue-specific manner (Peer et al., 2004; Peer and Murphy, 2006, 2007; Kuhn et al., 2011; Buer et al., 2013). Kuhn et al. (2017) showed that flavonol (a subgroup of flavonoids) accumulation in the *rol1-2* mutant, which is deficient in rhamnose synthase, affected shoot development and altered export of naphthalene-1-acetic (NAA), but not of IAA. Many reports suggest that flavonoids affect auxin transport directly or indirectly by modifying vesicular trafficking and PIN cycling, other auxin efflux proteins like ATP-binding cassette transporter superfamily (ABCB), or by modifying activities of PAT regulators, or protein phosphatase 2A (PP2A) and its antagonist PID (Peer et al., 2004; Terasaka et al., 2005; Geisler and Murphy, 2006; Kuhn et al., 2017). Clearly flavonoids are modulators of PAT, however, anthocyanins seem to have little or no effect on auxin transport (reviewed in Peer and Murphy, 2007). Interestingly, auxin accumulation is shown to promote flavonoid accumulation, presumably to quench ROS signal generated during auxin catabolism (Peer and Murphy, 2007; Peer et al., 2011). Similarly, enhanced flavonoid synthesis in auxin accumulating *PID^OE^* lines could also help to scavenge elevated ROS molecules. Another interesting assumption is that gradual accumulation of flavonoids in the leaves of *PID^OE^* lines further promotes localized auxin accumulation in the leaves, which is evident in both P10 and P21 (**Figures 1C, 5B, 7**).

In addition to increases in molecular antioxidants, also the activity of a relatively large range of antioxidant enzymes is, at least moderately, modified. The decreased activity of enzymatic antioxidants such as peroxidases, peroxiredoxins could also be seen as contributory factor in ROS accumulation in *PID^OE^* lines. The comparison of changes at the level of transcripts and enzyme activity is difficult to establish or rather inconclusive as most of redox-related genes were downregulated (Supplementary Table 3). The observation that a relatively large number of antioxidant enzymes and molecules change (mostly increase), suggest that a wide range of cellular redox processes are affected in the *PID^OE^* lines, rather than any specific process. The fact that changes in specific metabolites (anthocyanins) and enzymes (GO) are closely related to *PID* overexpression levels, indicates a positive correlation between them. We believe that the differences between two overexpression lines itself are because of the differences in *PID* expression levels and thus auxin levels and the fact that even transient changes in auxin can hugely impact cellular and molecular events in the plant (Nemhauser et al., 2006; Paponov et al., 2008; Saini et al., 2017). These data clearly indicate the presence of enhanced (oxidative) stress responses, in *PID^OE^* lines compared to *pid* knockouts, as shown by measurements of ROS, ROS producing and detoxifying enzymes, MDA and antioxidant molecules. To summarize, it is well established that environmental stresses impact cellular ROS levels, antioxidant concentration and their redox state (Mittler, 2002; Apel and Hirt, 2004; Hong-bo et al., 2008). Here, we demonstrate that in the absence of external stress, ROS and antioxidants levels change in response to cellular auxin perturbations, and modulate growth and stress adaptive responses.

### *PID* Overexpression Does Not Confer Tolerance to Water Stress

There are many reports providing links between abiotic stress, antioxidant abundance and auxin (Jung and Park, 2011; Hasanuzzaman et al., 2013; Rahman, 2013; Avramova et al., 2015, 2017). For example, activation of YUCCA7 elevates IAA levels and enhances resistance to drought in Arabidopsis (Lee et al., 2012). However, as a result of the overexpression of this gene, growth was reduced and plants had narrow and curled leaves. Similarly, overexpression of *OsPIN3t* increases water tolerance in rice (Zhang et al., 2012). *Phot1* (Wan et al., 2012), a close homolog of PINOID belonging to the same AGC kinase family (Galván-Ampudia and Offringa, 2007), is also shown to improve drought tolerance in Arabidopsis seedlings (Galen et al., 2007). Similarly, overaccumulation of flavonoids is shown to enhance tolerance to drought and osmotic stress (Nakabayashi et al., 2014), and anthocyanins are also suggested to have an osmoprotectant role among others, such as ROS scavenging (Hughes et al., 2010, 2013).

In our results, all three genotypes responded to osmotic stress by reducing their rosette growth (**Figure 6**). The differences in growth reduction between *PID^OE^* and WT could be due to already smaller size of non-stressed *PID^OE^* rosettes that allowed a lower degree of growth reduction when subjected to osmotic stress conditions. Both *PID^OE^* lines showed lower survival than the WT during drought stress assays in the soil. We believe that the increased IAA levels, due to *PID* overexpression, already induce stress conditions in the rosettes and that the water deficit treatment is additive, thereby leading to higher lethality. Another explanation could be that *PID^OE^* lines have much shorter roots and reduced numbers of lateral roots (Benjamins et al., 2001), making their quest for water in the soil very difficult, which could have resulted in poor performance under drought stress. Evidently, the shoot/root ratio and stress responses are closely related (Woo et al., 2007; Werner et al., 2010). This suggests that PID-induced auxin alterations induce the observed stress responses and clearly do not confer advantage in water deficit conditions.

To conclude, various reports show that plant AGC kinases participate in the response to biotic and abiotic stresses. We demonstrated that ectopic expression of *PID* causes changes in auxin levels and its response, and consequentially ROS is generated and other hormones are affected which results in the generation of growth inhibitory stress responses. Additionally, in contrast to several previous reports, our study uniquely shows that despite their high auxin and antioxidant levels, plants are impaired in their tolerance to osmotic and drought stress and show a low survival under severe drought conditions. For future work it will be interesting to know how direct the relation between PID and stress responses is and whether auxin accumulation in leaves is necessary or sufficient to cause the observed stress responses.

## Author Contributions

KS planned and performed most of the experiments, analyzed the data and wrote the article with contribution from HAs, EP, GB, and KV; HAb and SS provided assistance with data analysis; MM performed next generation sequencing; EP, GB, and KV conceived the project and KV supervised the research.

## Conflict of Interest Statement

The authors declare that the research was conducted in the absence of any commercial or financial relationships that could be construed as a potential conflict of interest.

## Footnotes

1 http://rsbweb.nih.gov/ij/

2 http://www.arabidopsis.org/

3 www.arabidopsis.org

4 https://www.r-project.org/
