# Supplementary material for: Perturbation of Auxin Homeostasis and Signaling by PINOID Overexpression Induces Stress Responses in Arabidopsis
Source: Front Plant Sci. 2017 Aug 2;8:1308. doi: 10.3389/fpls.2017.01308 (PMC5539238; doi:10.3389/fpls.2017.01308)
Supplement: Supplementary file 4 [file Image_1.pdf]

Supplementary Material

Perturbation of auxin homeostasis and signaling by *PINOID* overexpression induces stress responses in Arabidopsis.

Kumud Saini, Hamada AbdElgawad, Marios Nektarios Markakis, Sébastjen Schoenaers, Han Asard, Els Prinsen, Gerrit T.S. Beemster, Kris Vissenberg*

***Correspondence: Kris Vissenberg:** kris.vissenberg@uantwerpen.be

1. Supplementary Material and Methods

Hormone measurements

Extraction of acidic hormones

Plant material was homogenized by grinding and extracted overnight in 80% MeOH (10µl/mg FW, -20°C, 16h). C_6^{13}-phenyl-IAA (100 pmol, Cambridge Isotope Laboratories Inc., Andover, Massachusetts, USA), D_6-ABA (150 pmol, $[^2H_6]$(+)-*cis,trans* ABA (OlChemIm, Olomouc, Czech Republic), 100 pmol $[^2H_4]$-SA (OlChemIm), 100 pmol dehydro-Jasmonic acid (OlChemIm), 20 pmol $[^2H_2]$Gibberellin A1 (OlChemIm), 20 pmol $[^2H_2]$Gibberellin A3 (OlChemIm), 20 pmol $[^2H_2]$Gibberellin A4 (OlChemIm), 20 pmol $[^2H_2]$Gibberellin A7 (OlChemIm), 20 pmol $[^2H_2]$Gibberellin A8 (OlChemIm), 20 pmol $[^2H_2]$Gibberellin A9 (OlChemIm), 20 pmol $[^2H_2]$Gibberellin A12 (OlChemIm) and 20 pmol $[^2H_2]$Gibberellin A19 (OlChemIm) were added as internal standard. After a purification step removing pigments on a C18 cartridge (Bond Elut C18 6 cc, 500 mg, Agilent, CA, USA) in 80%MeOH, the extract is diluted and acidified with formic acid 6% so that hormones bind to a C18 cartridge. Diethyl ether is used to elute the hormones. The residual water is removed and the ether phase is evaporated under a stream of N_2 gas (Turbovac LV Evaporator, Hopkinton, MA, USA). After methylation with diazomethane (Schlenk and Gellerman, 1960), the samples were dried under N_2 gas, and dissolved in 100% hexane (15 µL) for GC-MS/MS analysis of SA and JA. The remaining of the samples after JA and SA analysis was dried and redissolved in 50 µl 10% MeOH for the LC-MS/MS analysis of IAA, ABA and gibberellins.

Quantification of SA and JA

Gas chromatography-mass spectrometry analysis was performed using a Waters Micromass Quattro micro GC (Waters; MA, USA): a triple quadrupole with an integrated Agilent 6890N gas chromatography oven, and using an electron impact (EI) ion source, positive ion mode, 70eV, Collision Energy 10 eV, inter-channel delay 10ms and inter-scan delay 10 ms. The gas chromatography column used was a 15 m x 0.25 mm Agilent J&W DB-5ms, film thickness 0.25 µm (Agilent Technologies; CA, USA), injection volume 10 µl, Carrier gas helium, flow rate 1 ml min^{-1}. The oven started isothermally at 50°C for 2 minutes, increased linearly to 300 °C at a rate of 25 °C

min^{-1}. 300 °C was held for 3 minutes. The diagnostic ions used for quantification of SA and JA in MRM mode are 152>120 m/z for MeSA, 156>124 m/z for D4-MeSA, 224>151 m/z for MeJA and 226>153 m/z for DHMeJA (dwell time 0.010 sec).

Quantification of IAA, ABA and GA

Gibberellins, IAA and ABA were analysed by UPLC-MS/MS after methylation (Acquity TQD, Waters, Manchester, UK) (6 µl injection by partial loop, ACQUITY BEHC18, 1.7 µm column (WATERS), column temp. 30°C, flow 400, solvent gradient 0-2 min: 95/5; 10% MeOH in NH4OAc 1 mM/MeOH; 2-4 min linear gradient until 10/90 10% MeOH in NH4OAc 1 mM/MeOH; 4-6 min, isocratic 10/90 10% MeOH in NH4OAc 1mM/MeOH; MS conditions: Polarity MS ES(+), capillary 2 kV, cone 20V, collision energy: 20 eV, source temperature: 120°C, desolvation Temperature: 450°C, Cone gas flow 50l/h, desolvation gas flow: 750l/h, collision gas flow: 0.19 ml/min). The diagnostic ions used for quantification for IAA and ABA are: 190>130 m/z for Me-IAA, 196>136 m/z for Me-C^{13}-IAA, 279>173 m/z for Me-ABA and 285>179 m/z for d_6-Me-ABA (dwell time 0.020sec). The diagnostic ions used for quantification for the gibberellins are : 382>333 m/z for d_2-GA1-Me, 380>331 m/z for GA1-Me, 380>241 m/z for d_2-GA3-Me, 378>239 m/z for GA3-Me, 366>317 m/z for d_2-GA4-Me, 364>315 m/z for GA4-Me, 362>285 m/z for GA5-Me, 364>315 m/z for d_2-GA7-Me, 362>313 m/z for GA7-Me, 398>349 m/z for d_2-GA8-Me, 396>347 m/z for GA8-Me, 350>301 m/z for d_2-GA9-Me, 348>299 m/z for GA9-Me, 399>300 m/z for d_2-GA12-Me, 397>298 m/z for GA12-Me, 393>300 m/z for D_2-GA19-Me, 391>298 m/z for d_2-GA19-Me and 364>315 m/z for GA20-Me (dwell time 0.010 sec). Methanol and water used for MS are UPLC grade from Biosolve (Valkenswaard, the Netherlands). Data are expressed in pmole per gram fresh weight (pmol.g^{-1}FW).

Extraction of neutral hormones (cytokinins)

Plant material was homogenized by grinding and extracted overnight in 80% MeOH (10µl/mg FW, -20°C, 16u). [$^2H_7$]N^6-Benzyladenine (d_7-BA), [$^2H_7$]N^6-Benzyladenosine (d_7-BAR), [$^2H_7$]N^6-Benzyladenine-*9*-Glucoside (d_7-BA*9*G), [$^{15}N_4$]*meta*-Topolin (^{15}N-*m*T), [$^{15}N_4$]*ortho*-Topolin (^{15}N-*o*T), [$^2H_3$]Dihydrozeatin (d_3-DHZ), [$^2H_3$]Dihydrozeatin Riboside (d_3-DHZR), [$^2H_6$]N^6-*iso*pentenyladenine (d_6-iP), [$^2H_6$]N^6-*iso*pentenyladenosine (D-*i*PR), [$^2H_5$]*trans*-Zeatin-*7*-Glucoside (d_5-*tZ7*G), [$^2H_5$]*trans*-Zeatin-*9*-Glucoside (d_5-*tZ9*G), [$^2H_5$]*trans*-Zeatin-*O*-Glucoside (d_5-*tZO*G), [$^2H_5$]*trans*-Zeatin-*O*-Glucoside Riboside (d_5-*tZ*R*O*G), [$^2H_6$]N^6-*iso*pentenyladenine-*7*-Glucoside(d_6-*i*P*7*G) and [$^2H_6$] N^6-*iso*pentenyladenine-*9*-Glucoside (d_6-*i*P*9*G) (10 pmol each, OlChemIm) were added as internal standard. After centrifugation (20000g, 15min, 4°C, 5810R, rotor FA-45-30-11 Eppendorf, Hamburg, Germany) the supernatant is passed over a C18 cartridge (500 mg, Varian) to retain pigments and consecutively filtered (Chromafil Xtra PA-20/25, 0.20µm, *Φ* 25 mm, MN, Düren, Germany). Samples were dried in a speed-vac (Christ RNC2-25 vacuum concentrator, with KNF N860.3FT.40.18 pump, Freiburg, Germany), and redissolved in 50 µl 10% MeOH for analysis.

Cytokinin quantification

Isoprenoid cytokinins were analysed by UPLC-MS/MS (WATERS) (6 µl injection by partial loop, ACQUITY BEHC18, 1.7 µm column (WATERS), column temp. 30°C, flow 400, solvent gradient 0-0.5 min: 95/5; 10% MeOH in NH4OAc 1 mM/MeOH; 0.5-3 min linear gradient until 75/25 10% MeOH in NH4OAc 1 mM/MeOH; 3-5 min, isocratic 75/25 10% MeOH in NH4OAc 1mM/MeOH;

5-6 min, linear gradient until 5/95 10% MeOH in NH4OAc 1 mM/MeOH; 6-6.5 min isocratic 5/95 10% MeOH in NH4OAc 1mM/MeOH; MS conditions: Polarity MS ES(+), capillary 2 kV, cone 20V, collision energy: 20 eV, source temperature: 120°C, desolvation Temperature: 400°C, Cone gas flow 20l/h, desolvation gas flow: 800l/h, collision gas flow: 0.22ml/min).

The diagnostic ions used for quantification for the isoprenoid cytokinins are : 225>136 m/z for d$_3$-DHZ, 222>136 m/z for DHZ, 220>136 m/z for Z, 357>225 m/z for d$_3$-DHZR, 354>222 m/z for DHZR, 352>220 m/z for ZR, 372>210 m/z for d$_6$-*i*P, 366>204 m/z for *i*P, 342>210 m/z for d$_6$-*i*PR, 336>204 m/z for *i*PR, 387>225 m/z for d$_5$-Z*7*G, d$_5$-Z*9*G, and d$_5$-Z*O*G, 384>222 m/z for DHZ*7*G, DHZ*9*G, and DHZ*O*G, 382>220 m/z for Z*7*G, Z*9*G, and Z*O*G, 519>387 m/z for d$_5$-*t*ZR*O*G, 516>384 m/z for DHZR*O*G, 514>382 m/z for ZR*O*G, 372>210 m/z for d$_6$-*i*P*7*G and d$_6$-*i*P*9*G, 366>204 m/z for *i*P*7*G and *i*P*9*G.

Aromatic cytokinins were analysed by UPLC-MS/MS (WATERS) (6 µl injection by partial loop, ACQUITY BEH C18, 1.7 µm column (WATERS), column temp. 40°C, flow 550, solvent gradient 0-1 min: 100/0; 10% MeOH in NH4OAc 1 mM/MeOH; 1-5 min linear gradient until 72/28 10% MeOH in NH$_4$OAc 1 mM/MeOH; 5-5.5 min, linear gradient until 0/100 10% MeOH in NH$_4$OAc 1mM/MeOH; min, 5.5-6 min isocratic 100%MeOH; MS conditions: Polarity MS ES(+), capillary 1.78 kV, cone 20V, collision energy: 12 eV, source temperature: 120°C, desolvation Temperature: 400°C, Cone gas flow 20l/h, desolvation gas flow: 800l/h, collision gas flow: 0.22ml/min).

The diagnostic ions used for quantification for the aromatic cytokinins are : 233>98 m/z for d$_7$-BA, 226>91 m/z for BA, 365>233 m/z for d$_7$-BAR, 358>226 m/z for BAR, 395>233 m/z for d$_7$-BA*9*G, 388>226 m/z for BA*9*G, BA*7*G and BA*3*G, 246>107 m/z for ^{15}N-*m*T, 242>107 m/z for *m*T, 247>141 for ^{15}N-*o*T, 242>136 m/z for *o*T and *p*T, 256>121 for Me-*m,o*-T, 374>242 m/z for *o,m,p*-TR, 404>242 m/z for *o,m,p* -T-G and 418>256 m/z for Me*m*T-G. Methanol and water used for MS are UPLC grade from Biosolve. Data are expressed in pmol per gram fresh weight (pmol.g^{-1}FW).

2. Supplementary data

Supplementary Data 1 RNA sequencing data for all the genes for which expression was detected in all three replicates of at least one time point and genotype i.e. WT and two *PID* overexpression lines.

Supplementary Data 2 RNA sequencing data for all 3805 differentially expressed genes in at least one timepoint and genotype i.e. WT and two *PID* overexpression lines.

Supplementary Data 3 List of all the overrepresented genes out of total 3805 differentially expressed genes between WT and two *PID* overexpression lines indicated in Figure 2B

3. Supplementary Figures and Tables

3.1 Supplementary Figures

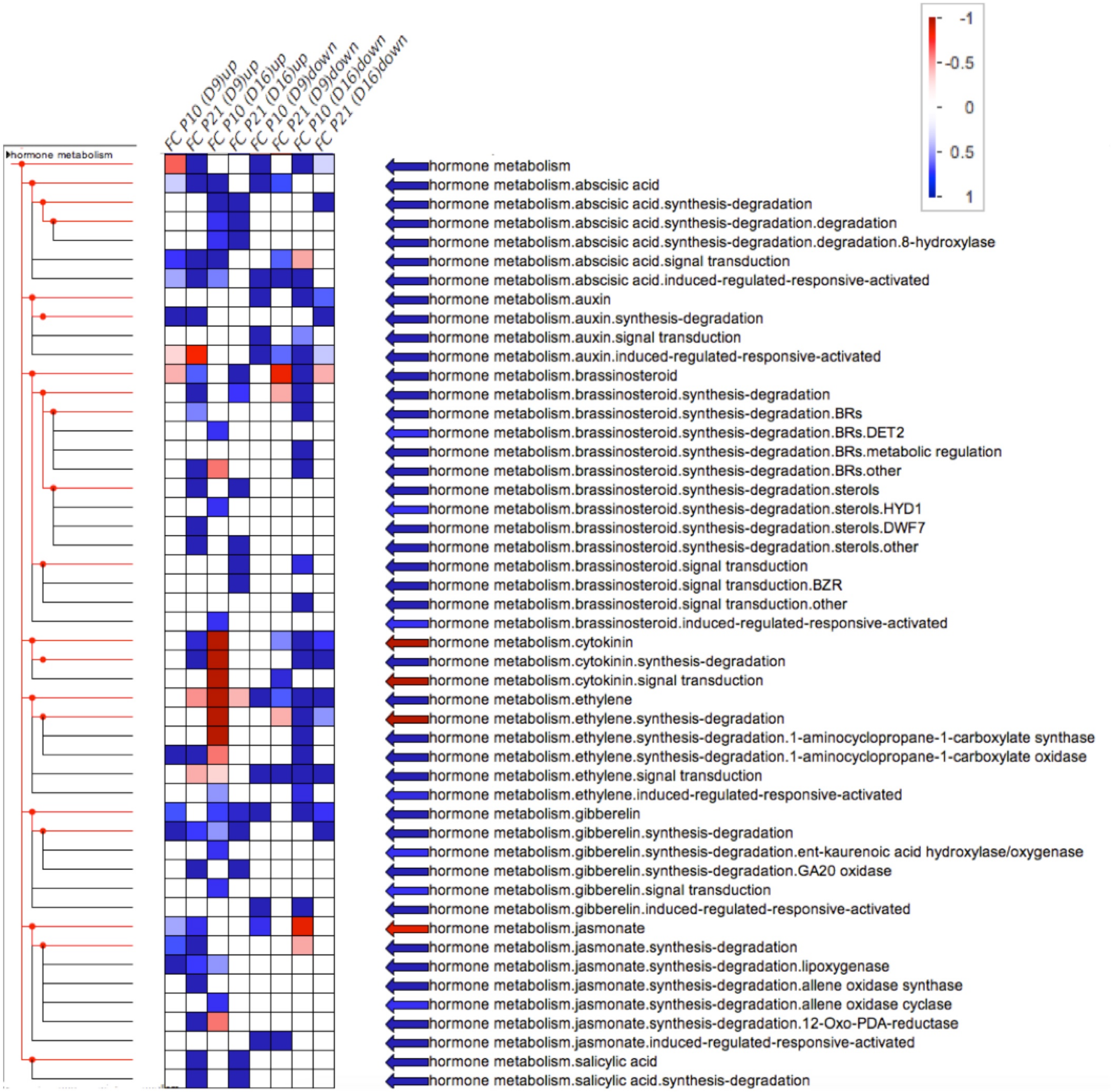

Supplementary Figure 1 PageMan representation of differentially expressed genes related to hormone metabolism in *PID*^{OE} lines vs the WT. PageMan representation of overrepresented biological pathways related to hormone metabolism (highlighted in blue) in *PID* overexpression lines at different time points that are up- and downregulated as indicated in the legend above. Pathways highlighted in red are underrepresented categories. FC=Fold change.

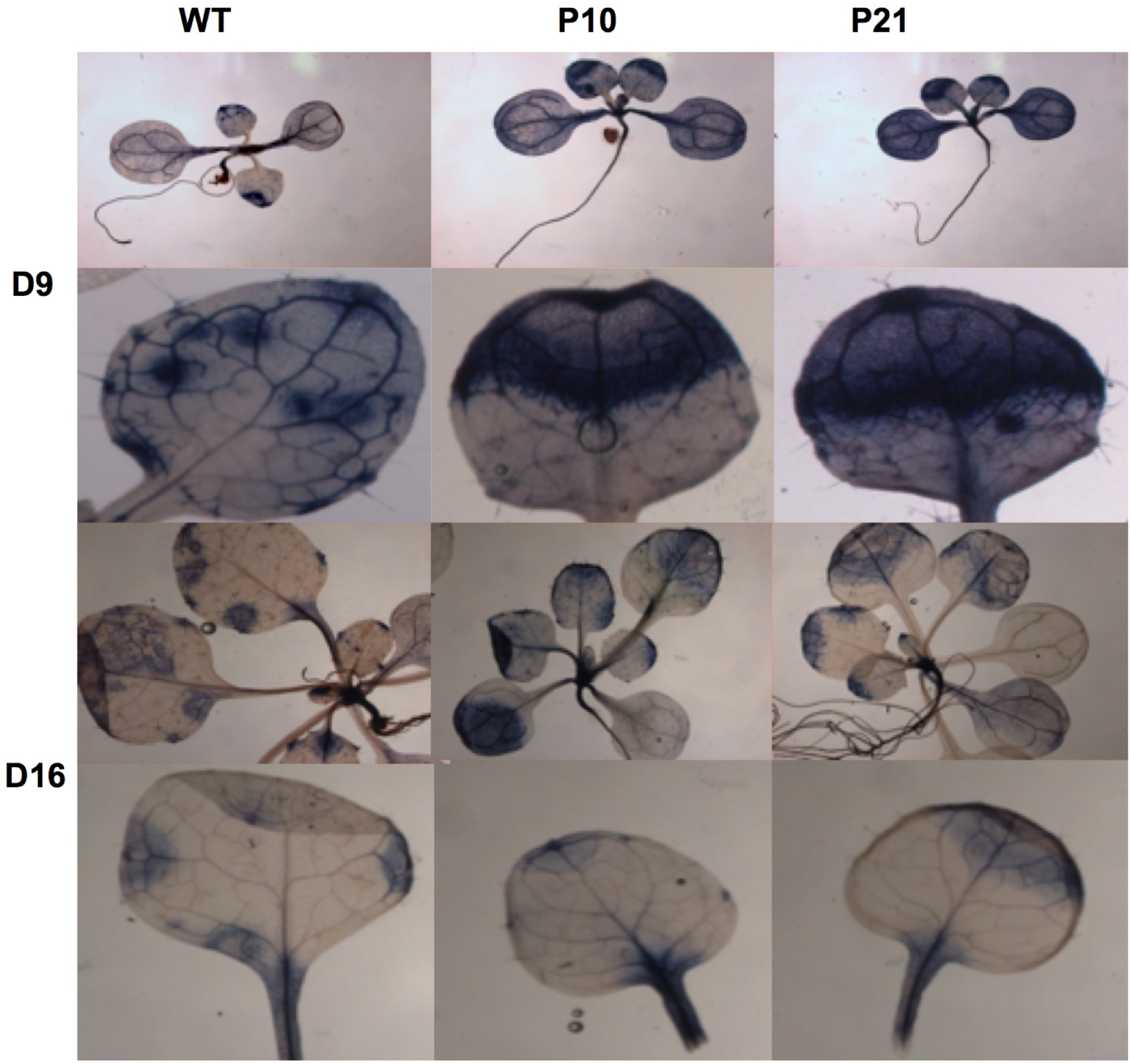

Supplementary Figure 2 Superoxide staining in rosettes and individual leaves of WT and *PID* overexpression lines. Visualization of the NBT (nitroblue tetrazolium) assay in the entire rosette and detached leaves of WT and both PID^{OE} lines at 9 and 16 DAS shows superoxide as blue color. Pictures are representative of the experiments, which were repeated twice.

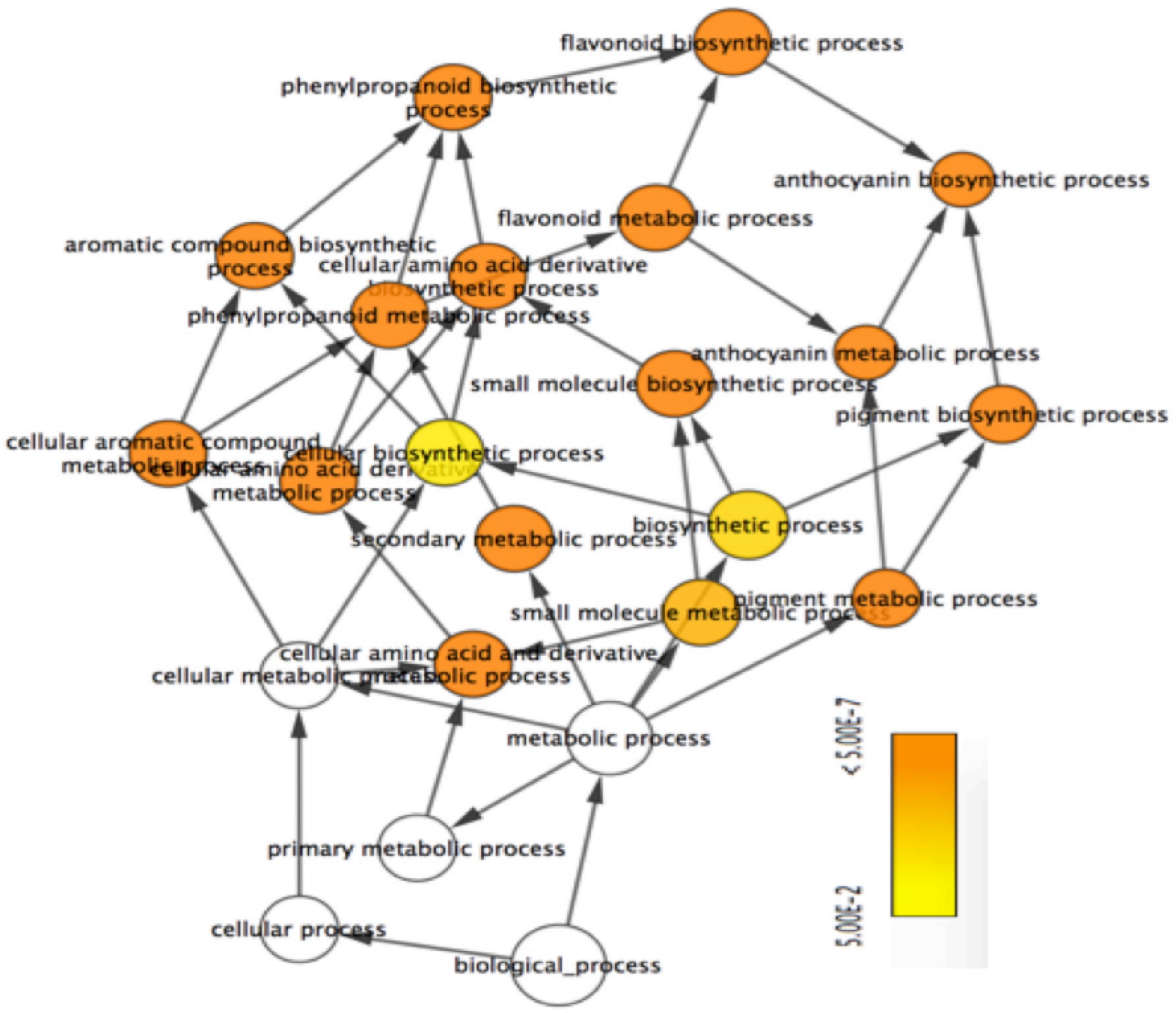

Supplementary Figure 3 Gene enrichment analysis showing overrepresentation of differentially expressed flavonoid-related genes in *PID*^{OE} lines.

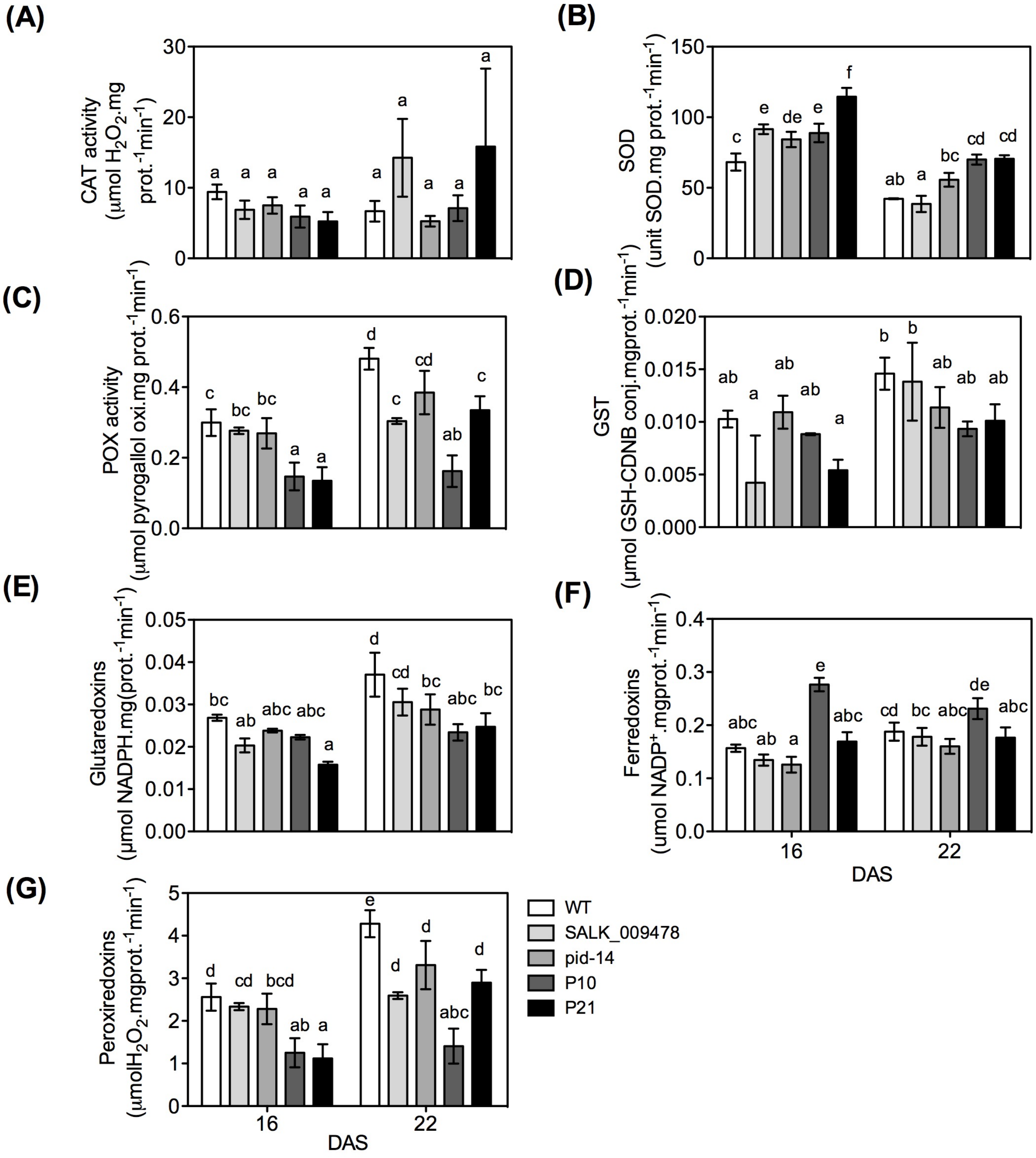

Supplementary Figure 4 Antioxidant enzymes profiling in the WT and *pid* knockouts and overexpression lines. Catalase; CAT (A) Superoxide dismutase; SOD (B). Peroxidases; POX (C). Glutathione-S-transferase; GST (D). Glutaredoxins (E). Ferroredoxins (F). Peroxiredoxins (G). Different letters in the graph represent significant differences between genotypes and time. (Duncan test; $P < 0.05$; n=3; Error bars: ± SE).

3.2 Supplementary tables

Supplementary Table 1 Primer sequences

Actin8	TGCTGGTCGTGACCTTACTG	CAATTTCCCGTTCTGCTGTT
PID intron spanning	GAGCAGAGATGGAGAAAACGA	TGAAAATGCTTGACCATCCA

Supplementary Table 2 Changes in expression levels of characteristic hormone-responsive genes in PID^{OE} lines. Significant $\log_2$ fold changes are in bold. Parentheses denote days after stratification. Abbreviations: Indole-3-acetic acid (IAA), Abscisic acid (ABA), Gibberellic acid (GA), Brassinosteroids (BR) Methyl jasmonate (MJ) and Cytokinins (CK).

	Feature ID	NAME	P10(9)	P21 (9)	P10 (16)	P21(16)
IAA	AT2G22810	ACCS4	0.264	-0.206	**-1.724**	-0.288
	AT3G15540	IAA19	1.204	**1.323**	**2.310**	0.237
	AT4G14560	IAA1	**1.191**	0.670	0.716	-0.257
	AT4G38860	SAUR16	-0.285	-0.001	**-0.874**	-0.418
	AT5G37770	TCH2	-0.420	0.119	**-1.367**	-0.153
	AT5G47370	HAT2	0.240	0.330	**1.415**	-0.369
	AT5G54510	GH3.6	**-2.042**	**-1.741**	-0.497	-0.003
	AT1G72430		-0.272	0.478	**-2.758**	-0.985

ABA	AT1G29395	COR414	1.128	1.215	**3.571**	-0.385
	AT1G32640	ATMYC2	0.542	-0.134	**1.831**	-0.051
	AT1G69260	AFP1	**-0.928**	0.207	**2.767**	-0.469
	AT1G69270	RPK1	0.392	0.356	**1.654**	-0.519
	AT2G22430	ATHB6	0.169	0.298	**1.280**	-0.069
	AT2G33380	RD20	1.396	3.264	**1.765**	-0.390
	AT2G46680	ATHB7	**1.668**	1.388	**3.651**	-0.438
	AT3G11410	PP2CA	0.340	0.449	**1.968**	-0.266
	AT3G14440	NCED3	0.495	0.345	**3.597**	-0.619
	AT3G15210	ERF4	**0.874**	0.326	**2.185**	0.081
	AT3G19290	ABF4	-0.057	0.254	**1.558**	-0.066
	AT3G61890	ATHB12	0.086	0.544	**2.226**	-0.210
	AT4G05100	MYB74	-0.422	1.364	**2.471**	-1.749
	AT4G26080	ABI1	0.250	1.095	**1.859**	-0.279
	AT4G34000	ABF3	0.542	**1.517**	**1.464**	-0.394
	AT5G57050	ABI2	0.429	1.050	**2.469**	-0.216
	AT5G59220	HAI1	-0.594	1.208	**4.485**	0.310
	AT5G59310	LTP4	1.557	**3.350**	0.661	0.000
	AT5G59320	LTP3	-1.845	0.148	**3.475**	-1.038
	AT5G66400	RAB18	0.444	1.842	**1.888**	-1.042
	AT2G15970	COR413	0.313	0.151	**1.674**	-0.412

	AT4G15910	DI21	**-1.068**	**-1.364**	**2.044**	0.633
	AT5G25610	RD22	**0.955**	**1.400**	**0.930**	0.066
	AT5G52300	RD29B	-0.288	-0.332	**7.802**	-0.511
	AT5G52310	RD29A	0.969	**1.278**	0.482	**-1.542**
	AT1G76180	ERD14	0.478	**0.874**	**1.205**	-0.569
GA	AT1G66350	RGL1	-0.337	-0.036	**1.507**	-0.898
BR	AT2G26710	BAS1	-0.092	0.078	**-1.372**	0.379
	AT1G13260	RAV1	0.033	-0.573	**-2.998**	-1.132
	AT3G13730	CYP90D1	0.254	0.637	**-1.319**	-0.542
	AT3G30180	CYP85A2	0.465	**1.023**	**-1.514**	-0.282
	AT3G61460	BRH1	0.659	**0.751**	**1.114**	0.048
	AT4G30610	BRS1	-0.211	0.065	**-1.561**	0.102
MJ	AT1G19570	DHAR1	0.426	**0.953**	**1.632**	-0.324
	AT1G32640	ATMYC2	0.542	-0.134	**1.831**	-0.051
	AT1G55020	LOX1	0.043	0.337	**1.222**	0.228
	AT2G34810		0.328	**1.153**	0.543	-0.126
	AT3G15210	ERF4	**0.874**	0.326	**2.185**	0.081
	AT3G45140	LOX2	**1.332**	**1.860**	**1.393**	-0.512
	AT4G23600	CORI3	**1.248**	**2.255**	0.171	-1.067

	AT5G42650	AOS	**0.944**	**1.212**	0.593	-0.519
	AT5G55120	VTC5	0.110	0.168	**0.976**	-0.097
	AT2G39940	COI1	0.209	0.195	**0.887**	-0.021
CK	AT1G03850	GRXS13	-0.519	-1.087	-3.445	-2.558
	AT1G10470	ARR4	-0.341	**-1.347**	-1.318	-0.130
	AT1G19050	ARR7	**-0.805**	**-2.764**	-1.471	0.497
	AT1G69040	ACR4	-0.244	-0.816	-1.424	-0.336
	AT1G74890	ARR15	-0.431	**-2.981**	-4.254	0.013
	AT2G01830	AHK4	-0.634	**-1.254**	-0.214	**0.966**
	AT3G48100	ARR5	-0.650	-2.945	-3.678	0.337
	AT5G62920	ARR6	-0.531	**-3.079**	-1.564	0.093
	AT1G27320	AHK3	0.454	**0.828**	0.516	0.034

Supplementary Table 3 Changes in expression levels of redox related and ROS producing genes in *PID*^{OE} lines. Significant log_2fold changes are in bold. Parentheses denote days after stratification.

Function	Feature ID	Name	P10 (9)	P21 (9)	P10 (16)	P21 (16)
ROS producing	AT3G14130	glycolate oxidase putative	0.138	0.273	**2.925**	1.176
	AT3G14150	glycolate oxidase putative	0.023	0.055	**1.755**	0.243
	AT1G19230	stress.biotic.respiratory burst	**1.964**	1.256	1.489	-1.111
Redox related	AT4G25100	FE SUPEROXIDE DISMUTASE 1	-0.624	**-0.903**	**-2.294**	0.164

AT1G19570	DHAR1	0.426	**0.953**	**1.632**	-0.324
AT1G63940	MDHAR6	-0.164	-0.488	**-1.048**	-0.504
AT5G21105	L-ASCORBATE OXIDASE	0.290	**0.816**	0.331	0.166
AT4G08390	STROMAL APX	**-0.926**	**-1.855**	**-3.874**	0.085
AT5G21100	L-ASCORBATE OXIDASE	0.428	**0.822**	-0.537	-0.015
AT2G25080	GPX1	-0.234	-0.102	**-1.433**	-0.523
AT4G15680	Glutaredoxin GRXS4	-1.576	-1.281	**-6.232**	**-0.942**
AT3G62930	GRXS6	-0.153	-0.813	**-3.265**	-0.650
AT4G15670	GRXS7	-1.093	-1.214	**-5.646**	**-1.463**
AT4G15690	GRXS5	-2.276	-2.276	**-5.419**	-0.420
AT1G03850	GRXS13	-0.519	-1.087	**-3.445**	-2.558
AT3G62950	GRXC11	-1.367	-3.804	**-3.836**	-1.041
AT4G15700	GRXS3	-0.589	-1.238	**-4.325**	-0.671
AT4G15660	GRXS8	0.454	0.000	**-4.327**	-0.139
